# Decentralized HIV testing: comparing peer and mail-based distribution strategies to improve the reach of HIV self-testing among people who use drugs in Florida

**DOI:** 10.1186/s12954-024-01031-9

**Published:** 2024-06-17

**Authors:** William H. Eger, Alexa Mutchler, Tim Santamour, Shelby Meaders, Heather A. Pines, Angela R. Bazzi, Hansel E. Tookes, Tyler S. Bartholomew

**Affiliations:** 1 School of Social Work, San Diego State University, San Diego, CA USA; 2 School of Medicine, University of California San Diego, La Jolla, CA USA; 3 Florida Harm Reduction Collective, St. Petersburg, FL USA; 4 School of Public Health, San Diego State University, San Diego, CA USA; 5 Herbert Wertheim School of Public Health, University of California San Diego, La Jolla, CA USA; 6 Boston University School of Public Health, Boston, MA USA; 7 Department of Medicine, Miller School of Medicine, University of Miami, Miami, FL USA; 8 Department of Public Health Sciences, Miller School of Medicine, University of Miami, Miami, FL USA

**Keywords:** HIV/AIDS, HIV self-testing, People who use drugs, Harm reduction, Syringe services program

## Abstract

**Introduction:**

People who use drugs (PWUD) are at increased risk for HIV infection. HIV self-testing (HIVST) is a promising method for identifying new infections, but optimal distribution strategies remain understudied.

**Methods:**

To characterize PWUD by HIVST distribution strategy (peers vs. mail), we examined data from July 2022 to June 2023 collected from a real-world HIVST program led by the non-profit, Florida Harm Reduction Collective. We used descriptive statistics and Poisson regressions with robust error variance to compare those who received HIVST through peers or via mail by socio-demographics, Ending the HIV Epidemic (EHE) county designation, and HIV testing experience.

**Results:**

Among 728 participants, 78% received HIVST from peers, 47% identified as cisgender female, 48% as heterosexual, and 45% as non-White; 66% resided in an EHE county, and 55% had no HIV testing experience. Compared to those who received an HIV self-test from peers, those who received tests via mail were less likely to be cisgender male (vs. cisgender female; prevalence ratio [PR] = 0.59, 95% confidence interval [CI]: 0.43, 0.81), non-Hispanic Black (vs. non-Hispanic White; PR = 0.57, 95% CI: 0.36, 0.89) or from EHE counties (vs. non-EHE counties; PR = 0.33, 95% CI: 0.25, 0.44). Those who received tests via mail were also more likely to identify their sexual orientation as “Other/Undisclosed” (vs. straight/heterosexual; PR = 2.00, 95% CI: 1.51, 2.66).

**Conclusion:**

Our findings support the role of community-based HIVST distribution strategies in increasing HIV testing coverage among PWUD. Additional research could help inform the equitable reach of HIVST.

## Introduction

People who use drugs (PWUD) that are unregulated, such as fentanyl and methamphetamine, and share syringes, are at elevated risk for HIV infection [1–4]. Fentanyl, in particular, has been linked to increased injection frequency among people who inject opioids, which can increase the risk of transmission in the event of receptive syringe re-use from someone living with unsuppressed HIV [5, 6]. Additionally, stimulant use is associated with heightened sexual risk-taking (e.g., condomless sex with multiple partners) that can increase exposure to HIV [7, 8]. This combination of injection- and sexual-related risk behaviors causes a duality of HIV risk for PWUD that highlights the need for prevention strategies that can disrupt both transmission routes within the social networks of PWUD [9].

HIV testing is a key intervention prioritized by the United States’ Ending the HIV Epidemic (EHE) Initiative (under the “Diagnose” pillar) [10]. However, HIV testing coverage among PWUD remains low due to multiple factors, including high levels of substance use stigma and difficulty accessing healthcare services in traditional settings [11–13]. HIV self-testing (HIVST) is an evidence-based strategy that can overcome barriers to traditional clinic-based HIV testing for at-risk communities, including men who have sex with men (MSM) and transgender individuals [14, 15]. Evaluation research has shown that specific HIVST distribution strategies, such as by peers or through the mail, can promote the reach of HIVST and effectively increase HIV testing uptake in communities disproportionately affected by HIV [16–25]. Although the provision of HIVST through community-based organizations (including syringe services programs; SSPs) is a promising, feasible, and acceptable strategy for increasing HIV testing among PWUD [26–28], little research has investigated how to best expand the reach of HIVST for PWUD, particularly to individuals who do not have access to brick-and-mortar harm reduction services. Furthermore, there is scant research comparing the reach of peer- and mail-based HIVST distribution strategies in other communities [15, 29], and none, to our knowledge, has been conducted among PWUD. In the context of an innovative, decentralized HIVST program for PWUD in Florida, we aimed to characterize the socio-demographics, EHE counties, and HIV testing experiences of PWUD reached by peer- and mail-based HIVST distribution.

## Methods

### Overview

This evaluation study used routine program operation data from a decentralized HIVST program implemented by the Florida Harm Reduction Collective (FLHRC) between July 2022 and June 2023. The FLHRC is a statewide, non-profit organization based in St. Petersburg that supports harm reduction organizations across Florida. At the direction of their community advisory board, the FLHRC implemented a statewide HIVST program to increase access to HIV testing among PWUD regardless of their route of substance administration (i.e., the program was not limited to people who inject drugs). The program distributed FDA-approved, over-the-counter rapid HIVST kits (the OraQuick At-Home HIV test; OraSure Technologies, Inc.), which can be easily administered in community-based settings such as SSPs [30, 31]. To promote access to HIVST, the program used peer- and mail-based distribution strategies, as described below.

### Procedures

#### Peer-based distribution strategy

Approximately 10 peers with lived experience of substance use conducted outreach to distribute HIVST kits to PWUD in public parks, homeless encampments, and other venues where PWUD were known to spend time. However, the number of peers and venues varied between counties, and over time, as new peers were trained (or left), and people relocated or were involuntarily displaced by encampment sweeps [32]. Peers attended the Florida Department of Health’s 501/502 HIV training and were required to read and watch several training modules on HIV and the OraQuick At-Home HIV Test produced by the Centers for Disease Control and Prevention and OraSure Technologies Inc., respectively. Peers utilized engagement strategies, such as offering hygiene kits and condoms, to build trust and rapport with participants. If participants indicated an interest in HIVST, peers provided HIVST kits with informational postcards; acceptance of an HIVST kit was not required to receive engagement items. Participants could self-test immediately with the support of the peer distributor or take the kit with them to self-test later in private. If completed immediately, peers recorded test results and other relevant information (described below) using an anonymous HIPAA-compliant online survey delivered through a password-protected phone or laptop provided by the FLHRC (JotForm Inc., San Francisco, CA). Participants who chose to test later in private were given postcards with a QR code to an anonymous survey to report their results back to the FLHRC. Survey questions were consistent whether participants answered together with peers or in private.

#### Mail-based distribution strategy

The FLHRC also operates a robust mail-based naloxone distribution program that has been in operation since March 2022, which served as the primary marketing strategy for the mail-based HIVST kits. Postcards with information on how to order HIVST kits from the FLHRC were included in the naloxone kits. If participants chose to order HIVST kits, they could scan the QR code on the postcard or go directly to the FLHRC’s website to complete an anonymous survey (described below) and a separate form with mailing information for order fulfillment. In addition, the FLHRC collaborated with NEXT Distro, an online harm reduction organization that sends sterile syringes and other drug-use equipment, as well as naloxone, directly to PWUD [33, 34], where participants could access the link to FLRHC’s HIVST program.

Once order requests were received, the FLHRC sent an envelope packed with the following items: one HIVST kit; an informational postcard with a QR code to the FLHRC’s website; information on locally available resources participants could access depending on their test results (e.g., if non-reactive, information on HIV pre-exposure prophylaxis (PrEP) was provided); and another postcard with a QR code to a separate, optional anonymous survey to report their results back to the FLHRC. At the time of data collection, no contact information was collected on this optional follow-up survey for mail-based test recipients.

### Survey measures

Our primary aim was to compare participants who received an HIVST kit from a trained peer to those who received a kit via mail. Characteristics of interest included gender identity, sexual orientation, race, ethnicity, EHE county designation, and prior HIV testing experience. In the survey, participants (or peers, depending on distribution strategy) could select from the following gender identities: cisfemale, cismale, transwoman, transman, non-binary, genderqueer, intersex, two-spirit, agender, pangender, intersex, questioning, other, unknown, and prefer not to answer, which were later categorized as “Cis-Female,” “Cis-Male,” and “Other/Undisclosed” (i.e., transwoman, transman, non-binary, genderqueer, intersex, two-spirit, agender, pangender, intersex, questioning, other, unknown, and prefer not to answer). Sexual orientation options included: straight/heterosexual, gay/lesbian, bi-sexual, polyamorous, queer, asexual, pansexual, questioning, other, unknown, and prefer not to answer, which were then categorized as “Straight/Heterosexual,” “Gay/Lesbian,” or “Other/Undisclosed” (i.e., bi-sexual, polyamorous, queer, asexual, pansexual, questioning, other, unknown, and prefer not to answer). For ethnicity, participants were asked if they identified as either “Hispanic” or “non-Hispanic.” For race, participants were asked if they identified as “Black,” “Latinx,” “Native American/Indigenous,” “Asian,” “Asian Subcontinent,” “White/Caucasian,” “Other,” or “Prefer not to answer.” For analysis purposes, ethnicity and race were combined into the following categories: “Non-Hispanic White,” “Non-Hispanic Black,” “Hispanic/Latinx,” and “Other/Undisclosed.” As designated by the Centers for Disease Control and Prevention (CDC), EHE counties in Florida include the following seven jurisdictions: Broward, Duval, Hillsborough, Miami-Dade, Orange, Palm Beach, and Pinellas [35]. All other Florida counties were considered non-EHE. Lastly, participants were asked to indicate whether they had any experience with HIV testing, which was dichotomized into “Yes” or “No,” with “Unsure” responses categorized as missing.

### Statistical analysis

We calculated descriptive statistics to characterize our sample overall and by HIVST distribution strategy (peer- or mailed-based). To compare those reached by each HIVST strategy, we used Poisson regressions with robust error variances to model HIVST distribution strategy (peer- vs. mail-based) as a function of participants’ characteristics (i.e., socio-demographics, EHE county designation, and HIV testing experience). We fit separate Poisson regression models with robust error variances for each characteristic to obtain prevalence ratios (PRs) and corresponding 95% confidence intervals (CIs). All analyses were conducted using R version 4.1.2.

## Results

Among 728 total participants who were surveyed, 570 (78%) received an HIVST kit from a peer and 158 (22%) received an HIVST kit via mail. About half of participants identified as cisgender female (47%) and straight/heterosexual (48%). Most were non-Hispanic White (55%) and from an EHE county (66%). Importantly, most had no prior HIV testing experience (55%) and did not return their HIVST result (73%; Table 1). No participants receiving mail-based kits completed the optional follow-up survey to report their results. Therefore, all returned HIVST test results were from participants who received peer-distributed tests, with approximately 5% of these tests reported as reactive.

**Table 1:** Characteristics of participants accessing Florida’s HIVST Program by distribution strategy and unadjusted associations between distribution strategy and gender, sexual orientation, race/ethnicity, EHE county designation, and HIV testing experience (*N* = 728)

	HIVST Distribution Strategy						
	Mail (*n* = 158) *n* (%)^a^	Peer (*n* = 570) *n* (%)^a^		Total (*N* = 728) *n* (%)^a^		Mail vs. Peer PR (95% CI)^b^	
**Gender**							
Cis-Female	93 (58.9%)	249 (43.7%)		342 (47.0%)		Ref	
Cis-Male	46 (29.1%)	241 (42.3%)		287 (39.4%)		0.59 (0.43, 0.81)	
Other^c^	19 (12.0%)	44 (7.7%)		63 (8.7%)		1.11 (0.73, 1.68)	
Missing	0 (0%)	36 (6.3%)		36 (4.9%)		N/A	
**Sexual Orientation**							
Straight/Heterosexual	61 (38.6%)	287 (50.4%)		348 (47.8%)		Ref	
Gay/Lesbian	9 (5.7%)	40 (7.0%)		49 (6.7%)		1.05 (0.56, 1.97)	
Other^d^	88 (55.7%)	163 (28.6%)		251 (34.5%)		2.00 (1.51, 2.66)	
Missing	0 (0%)	80 (14.0%)		80 (11.0%)		N/A	
**Race/Ethnicity**							
Non-Hispanic White	99 (62.7%)	298 (52.3%)		397 (54.5%)		Ref	
Non-Hispanic Black	19 (12.0%)	115 (20.2%)		134 (18.4%)		0.57 (0.36, 0.89)	
Hispanic/Latinx	24 (15.2%)	76 (13.3%)		100 (13.7%)		0.96 (0.65, 1.42)	
Other^e^	16 (10.1%)	41 (7.2%)		57 (7.8%)		1.13 (0.72, 1.76)	
Missing	0 (0%)	40 (7.0%)		40 (5.5%)		N/A	
**EHE County Designation**							
Non-EHE County	95 (60.1%)	148 (26.0%)		243 (33.4%)		Ref	
EHE County^f^	63 (39.9%)	420 (73.7%)		483 (66.3%)		0.33 (0.25, 0.44)	
Missing	0 (0%)	2 (0.4%)		2 (0.3%)		N/A	
**Any HIV Testing Experience**							
Yes	46 (29.1%)	113 (19.8%)		159 (21.8%)		Ref	
No	99 (62.7%)	298 (52.3%)		397 (54.5%)		0.86 (0.64, 1.16)	
Missing	13 (8.2%)	159 (27.9%)		172 (23.6%)		N/A	
**HIVST Return Status**							
Not Returned	158 (100%)	378 (66.3%)		536 (73.6%)		N/A	
Returned	0 (0%)	192 (33.7%)		192 (26.4%)		N/A	
**HIV Test Result**							
Non-reactive	0 (0%)	166 (29.1%)		166 (22.8%)		N/A	
Reactive	0 (0%)	26 (4.6%)		26 (3.6%)		N/A	
Unsure/Unknown	0 (0%)	2 (0.4%)		2 (0.3%)		N/A	
Missing	158 (100%)	376 (66.0%)		534 (73.4%)		N/A	

^a^ Percentage (%) values may not add up to 100% due to rounding; Total column Ns may not sum to totals due to missing values

^b^ Prevalence ratios and 95% confidence intervals were estimated using Poisson regression with robust error variance with peer-distribution as the reference group

^c^ Defined as any of the following: trans-man, trans-woman, non-binary, genderqueer, intersex, two-spirit, agender, pangender, intersex, questioning, other, unknown, or prefer not to answer

^d^ Defined as any of the following: bi-sexual, polyamorous, queer, asexual, pansexual, questioning, other, unknown, or prefer not to answer

^e^ Defined as any of the following non-Hispanic races: Native American/Indigenous, Asian, Asian Subcontinent, Other, or Prefer not to answer

^f^ Includes Broward, Duval, Hillsborough, Miami-Dade, Orange, Palm Beach, and Pinellas Counties

Abbreviations: N/A = not assessed; EHE = Ending HIV Epidemic; HIVST = HIV self-test

Compared to those who received HIVST kits from peers, those who received tests via mail were less likely to be cisgender male (vs. cisgender female; PR = 0.59, 95% CI: 0.43, 0.81), non-Hispanic Black (vs. non-Hispanic White; PR = 0.57, 95% CI: 0.36, 0.89), or from an EHE county (vs. non-EHE county; PR = 0.33, 95% CI: 0.25, 0.44). Additionally, compared to those who received tests from peers, those who received tests via mail were more likely to identify their sexual orientation as “Other/Undisclosed” (vs. straight/heterosexual; PR = 2.00, 95% CI: 1.51, 2.66). HIV testing experiences did not differ between those who received tests from peers vs. mail (PR = 0.86, 95% CI: 0.64, 1.16).

## Discussion

We evaluated the first, to our knowledge, real-world implementation of HIVST for PWUD. HIVST is a promising intervention for overcoming barriers to traditional, clinic-based HIV testing for PWUD at risk for HIV infection [1–4], and could be particularly beneficial for our study population given that over half of our sample reported never being tested for HIV in the past. However, research on HIVST among PWUD remains scarce, and effective strategies for supporting the equitable delivery of HIVST to this community are understudied. Our findings highlight notable differences in the communities reached through peer- and mail-based distribution strategies, carrying implications for future HIVST program implementation for PWUD.

As noted above, over half of participants in this study reported that their HIVST kit from the FLHRC was the first HIV test they had ever received, and most resided in EHE counties, where HIV transmission is highest in the nation [35]. HIVST programs have historically reached individuals who have never or infrequently tested before but represent other key communities (i.e., MSM, transgender people, and female sex workers) for HIV prevention [14, 15]. A systematic review that compared HIVST to standard clinic-based HIV testing strategies among MSM and transgender individuals showed that HIVST increased testing uptake by 1.5 times and increased the mean number of HIV tests conducted by an organization by 2.6-fold over follow-up [15]. To our knowledge, this is the first study to highlight similar increases in HIV testing utilization for PWUD. Our findings support the need for further investment in HIVST for PWUD and its integration within harm reduction organizations such as SSPs. While SSPs initially evolved to address HIV transmission among PWUD at the onset of the HIV/AIDS epidemic in the U.S [36], funding and demand for HIV prevention within SSPs has diminished over time [37–39]. However, isolated HIV outbreaks among PWUD now threaten efforts to end the HIV epidemic and highlight the need for the implementation of additional HIV prevention services, including HIVST, within harm reduction spaces [40, 41].

We found that a greater proportion of participants who received an HIVST kit via mail identified as cisgender female, non-Hispanic Black, reported their sexual orientation as “Other/Undisclosed,” and were from non-EHE counties. Mail-based distribution of HIVST may offer a higher degree of privacy and convenience than facility-based HIV testing and peer-based distribution of HIVST, which may be more appealing to cisgender females, people who identify as non-Hispanic Black, and individuals whose sexual identities are often stigmatized in traditional healthcare settings [42, 43]. Mail-based distribution of HIVST can, therefore, circumvent the need for PWUD to decide between HIV testing and facing discrimination. PWUD with these socio-demographic characteristics and from non-EHE counties might face additional barriers to accessing traditional healthcare settings because of geographical location, lack of transportation, or limited opportunities caused by structural disparities in access to care. Because HIV prevention resources are limited and targeted to EHE counties [10, 44], mail-based distribution of HIVST could provide a solution for PWUD at risk for HIV in non-EHE designations without the need for in-person HIV prevention infrastructure (e.g., Ryan White clinics). In Florida, all legal operational SSPs are in EHE jurisdictions and offer opt-out HIV testing, likely decreasing the need for HIVST in these communities [45].

One of the primary challenges encountered by HIVST programs is the effective management of returning test results to organizations providing the tests. In our sample, no participant who received an HIVST kit by mail returned their HIV test result. Based on the broader literature, we can assume that the HIV positivity rates for both peer- and mail-based delivery strategies are comparable [14, 15], suggesting that approximately 5% of mail-based test users in our sample could have had a reactive result. From a public health perspective, the return of HIV test results is desirable as it facilitates referrals to appropriate HIV-related clinical services (for treatment or prevention) and supports the cascade of contact tracing. Yet, this imperative for result return could potentially clash with the anonymity of HIVST. While the absence of any results here was somewhat unexpected, it aligns with findings from a meta-analysis of HIVST across various key communities for HIV prevention (e.g., MSM and female sex workers), which revealed that HIVST is associated with a 17% reduction in linkage to care compared to standard facility-based HIV testing [15]. These dynamics underscore the complex trade-offs between anonymity and the public health imperative to facilitate follow-up care and support for individuals with reactive test results, highlighting the need for innovative HIV prevention approaches that may involve a blend of peer-facilitated mail-delivery of HIVST.

### Limitations

The implications of this work should be interpreted in the context of several limitations. First, our study considered a real-world HIVST program that was not implemented within a generalizable sample or location. Still, it provides an example of how an HIVST program for PWUD could function in other real-world settings. Similarly, the HIVST program was available to anyone accessing peer-based or online services offered by the FLRHC or NEXT Distro, meaning there were no strict eligibility criteria, and individuals who do not use drugs could have accessed the program. With that said, the FLHRC specifically trained peers to deliver services to PWUD, and NEXT Distro is a harm reduction organization known to serve this community. Moreover, there were no set schedules for outreach by trained peers across the FLHRC, which may have impacted the availability of services for specific communities. However, we do not anticipate this would have introduced differential bias by testing strategy. In addition, because the program was not created for research, our analyses could not include several characteristics that may also be associated with receipt of HIVST from a peer or via mail, such as housing status or income. Since the FLHRC’s HIVST program was anonymous there may be duplicates in our data, although the risk is low considering the timeframe of our assessment and the recommended frequency of HIV testing.

## Conclusion

This study underscores the potential of HIVST to increase testing uptake among PWUD, especially individuals without prior HIV testing experience and living in non-EHE jurisdictions where HIV prevention resources may be scarce. To maximize its public health impact, HIVST programs should tailor distribution strategies and follow-up supports to the specific needs and preferences of different communities of PWUD. Ultimately, HIVST holds promise to improve HIV detection and prevention efforts among PWUD, contributing to the broader goal of reducing incident HIV infections and ending the HIV epidemic in the United States. However, future research is needed to advance the equitable reach of HIVST, especially among communities of PWUD.

## Data Availability

No datasets were generated or analysed during the current study.

## Abbreviations

PWUD: People Who Use Drugs
HIV: Human Immunodeficiency Virus
EHE: Ending the HIV Epidemic
HIVST: HIV self-testing
SSP: Syringe Services Program
MSM: Men Who Have Sex with Men
FLHRC: Florida Harm Reduction Collective
FDA: U.S. Food and Drug Administration
PR: Prevalence Ratio
CI: Confidence Interval
AIDS: Acquired Immunodeficiency Syndrome
